# Long-term preservation of planar cell polarity in reversed tracheal epithelium

**DOI:** 10.1186/s12931-018-0726-y

**Published:** 2018-02-02

**Authors:** Takuya Tsuji, Ryosuke Nakamura, Tatsuya Katsuno, Yo Kishimoto, Atsushi Suehiro, Masaru Yamashita, Ryuji Uozumi, Tatsuo Nakamura, Ichiro Tateya, Koichi Omori

**Affiliations:** 1 Department of Otolaryngology-Head & Neck Surgery, Graduate School of Medicine, Kyoto University, 54 Kawahara-cho, Shogoin, Sakyo-ku, Kyoto, 606-8507 Japan; 2 Department of Biomedical Statistics and Bioinformatics, Graduate School of Medicine, Kyoto University, Kyoto, Japan; 3 Department of Regeneration Science and Engineering, Institute for Frontier Life and Medical Sciences, Kyoto University, Kyoto, Japan

**Keywords:** Planar cell polarity, Reversed trachea, Cilia

## Abstract

**Background:**

Planar cell polarity (PCP) coordinates the patterning and orientation of cells and their structures along tissue planes, and although its acquisition during the formation of airway epithelium has been described, the mechanisms for its maintenance and reconstruction are poorly understood. We aimed to clarify whether ambient environment change by orthotropic autologous transplantation affected PCP at the cellular level.

**Methods:**

We performed orthotropic autologous transplantation by inverting tracheal segments in rats, and then performed morphological evaluation by microscopy. The PCP of the tracheal epithelium was assessed over time by analyzing the directions of mucociliary transport and ciliary beat, the positional relationship between the basal body and basal foot, and the bias of Vang-like protein 1 (Vangl1) at 2, 4, and 6 months postoperatively.

**Results:**

After 2 months, the directions of mucociliary transport and ciliary beat were preserved toward the lung in the inverted tracheal segments. The positional relationship between the basal body and the basal foot, and the bias of Vangl1, also indicated preservation of PCP in the inverted tracheal segments. Similar results were obtained at 6 months.

**Conclusion:**

The PCP of ciliated epithelium was preserved in reversed trachea, even after long-term observation.

**Electronic supplementary material:**

The online version of this article (10.1186/s12931-018-0726-y) contains supplementary material, which is available to authorized users.

## Background

The inner airway surface is exposed to at least 10,000 L of air per day by breathing, the action of which introduces dust and microorganisms that may cause infection, inflammation, or obstruction if they are left to accumulate. Therefore, effective protection mechanisms have evolved to clean the airway and maintain homeostasis, with one of the most important being mucociliary clearance, which creates a fluid flow on the airway surface by coordinating mucus secretion and ciliary movement [1, 2]. In normal ciliated epithelia in the trachea, multiciliated cells are arranged so that mucus flow is toward the pharyngeal side, promoting the excretion of foreign materials by ordered ciliary motion. This directionality follows the planar cell polarity (PCP) of the airway epithelium [3–6].

PCP is a property that coordinates the patterning of cells and the orientation of their structures along tissue planes [7]. Such polarized structures exist in the bristles of insects, the stereocilia bundles of inner ear hair cells, and in the feathers, scales, and epidermal hairs of vertebrates [8–10]. The property is also apparent in the collective cell movements observed during tissue development [11–14]. Several important PCP signaling molecules in *Drosophila*, such as Frizzled and Vang, are also expressed in the tracheal tissue of mammals (e.g., the Frizzled and Vang-like [Vangl] families). In the ciliated cells of airway epithelia, proximal localization of Vangl and distal localization of Frizzled is necessary for ordered distal-to-proximal ciliary movement [15, 16]. The directionality of ciliary beating is determined by the positional relationship of the basal body and basal foot apparatuses at the base of each cilium [17–20].

In recent years, tracheal reconstruction has developed based on tissue engineering techniques [21–23] that use cell-free and cellularized scaffolds to reconstruct tracheal defect areas. Although ciliary beat frequency analysis has revealed that the restoration of normal cilia motility is achieved by a cell-free scaffold, the direction of ciliary movement remains to be investigated [21]. Scaffold coated with cultured ciliated epithelium is one option [24], but it is desirable for the epithelial cells to acquire coordinated PCP in culture before transplantation, or to adjust PCP after transplantation.

The process of PCP acquisition during the development and in vitro differentiation of airway epithelium has been reported [6, 15, 16, 25]. However, there is only limited research on how planar polarity is maintained in the airway epithelium during tissue turnover or regeneration. It has been reported that in tracheal epithelial cell culture, the localization of Vangl1, a PCP-related protein, is preserved in individual cells; however, the overall direction of the whole cell layer is disaggregated [16]. This suggests that external factors are required to maintain PCP coordination of airway epithelium, though there has been no reported success in inducing coordinated PCP by applying external shear stress to airway epithelial cell cultures [25]. The overall directional signal (analogous to the Fat/Dachsous mechanism in *Drosophila*) is not known in mammalian trachea [26, 27]. The conditions determining PCP in airway epithelial cells, therefore, remain unknown.

There is a question of whether it is possible to adjust PCP afterwards in regenerated tracheal epithelium once acquired. There have been no reports on whether tracheal epithelium can acquire or change PCP according to the surrounding in vivo environment. Interestingly, it has been reported that directional mucus flow was maintained after autologous in vivo transplantation of tracheal segments in dogs and rabbits [28, 29]. These studies showed that mucus in the inverted tracheal segments were transported from the pharyngeal to the lung side, providing evidence that airway epithelial tissue maintains planar polarity even if the surrounding environment changes. However, there is no evidence about the change in PCP at the cellular or molecular level, and the long-term effects in inverted tracheae are unknown.

In this study, we performed inverted tracheal autotransplantation in rats to improve our understanding of the in vivo formation and maintenance of PCP in airway epithelium. We examined whether the ambient environmental change induced by orthotropic reversing affected the airway epithelial planar polarity at the cellular and molecular levels.

## Methods

### Animals

We used 13 male 10-week-old Wistar/ST rats in this study. All experimental protocols were approved by the Animal Research Committee of Kyoto University Graduate School of Medicine. Animal care was provided by the Institute of Laboratory Animals of Kyoto University.

### Surgery and sampling

**Fig. 1:**
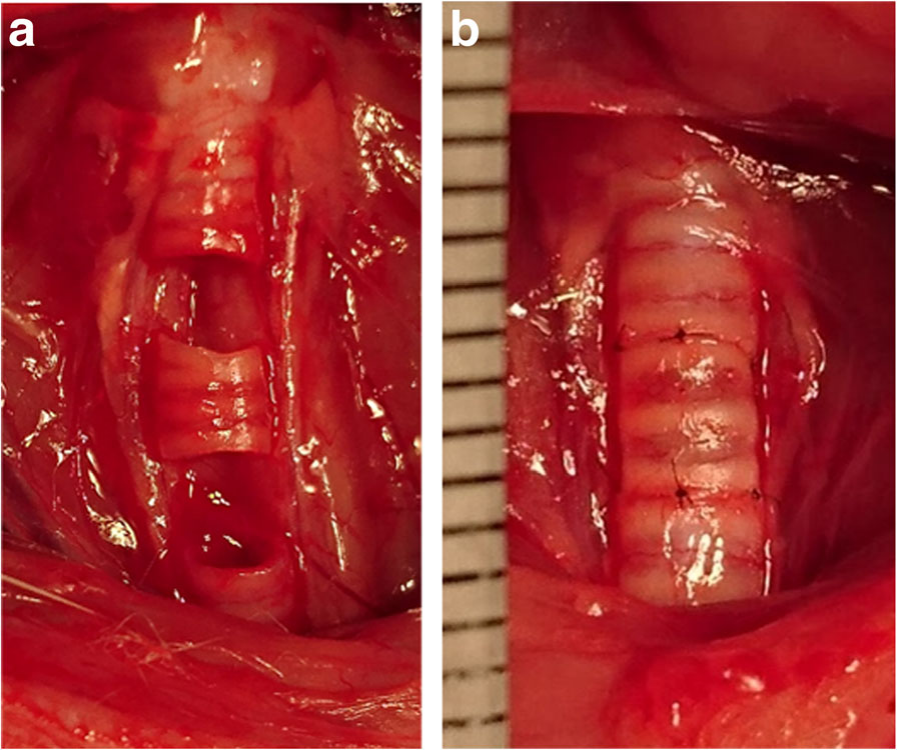
Autologous Transplantation of Inverted Tracheal Segments. **a** Tracheal segments containing three cartilages were resected. **b** The segments were reversed and anastomosed to the cut edges of normal tracheae

In all rats, surgery was performed under general anesthesia induced by intraperitoneal injection of ketamine (40 mg/kg) and xylazine (6 mg/kg). Under aseptic conditions, skin and subcutaneous tissues were incised and the submandibular glands and strap muscles were separated and retracted laterally to enable access to the trachea. While preserving the recurrent laryngeal nerve, the trachea was dissected from the esophagus and a tracheal segment containing three tracheal cartilages was removed (Fig. 1a). The extracted tracheal segment was inverted and replaced at the original site by end-to-end anastomosis with four 9–0 proline sutures. Subsequently, the wound was closed in anatomical layers using 3–0 Vicryl (Fig. 1b). To examine the acute changes to cilia and mucosa, one rat from each group was sacrificed 5 days after surgery. Three rats each were then sacrificed at 2, 4, and 6 months after surgery. A control group (*n* = 3) underwent tracheal removal and replacement without performing tracheal inversion, and the rats were sacrificed 2 months after surgery. In all cases, the normal upper, treated middle, and normal lower portions were harvested together. All rats survived the experimental period without notable cough, wheeze, or tachypnea.

### Analyses of Mucociliary transport and ciliary beat directions

Tracheae were cut along the long axis to obtain 2-mm wide strips of tracheae. They were fixed on 35-mm dishes with Vetbond (3 M, Maplewood, MN, USA) and monitored for mucociliary transport and ciliary movement. Fluorescent microspheres (Polysciences, Warminster, PA, USA) dissolved in Dulbecco’s Modified Eagle’s Medium (DMEM)/nutrient mixture F-12 (DMEM/F-12; Nacalai Tesque) was poured onto the luminal surfaces of tracheae. Movements of the microspheres were observed under an upright microscope (model BX51, Olympus, Tokyo, Japan) and recorded at 50 frames/s, using a high-speed camera (FASTCAM mini UX50; Photron, Tokyo, Japan). The directions of microsphere movements were measured between sequential frames by DIAtrack software (downloaded from http://www.diatrack.org/). To monitor ciliary movement, tracheal samples were reacted with 4 μg/mL fluorescein isothiocyanate-conjugated wheat germ agglutinin (FITC-WGA; Vector Laboratories, Burlingame, CA, USA) for 2 h to aid visualization. After rinsing with DMEM (Nacalai Tesque), the tracheae were laid in DMEM/F-12 and the lumen was observed with a BX-51 microscope. Ciliary movement was recorded at 125 frames/s and the direction of ciliary beat in at least 100 ciliated cells was analyzed, using ImageJ (National Institutes of Health, Bethesda, MD, USA). A Kruskal–Wallis test was performed to compare differences among the groups. The mean direction value of the control group was increased by 180 ° to calculate the *p*-value.

### Immunofluorescence microscopy

Tracheal samples obtained at 5 days and at 2 and 6 months after the operation were dissected and fixed with 10% (*w*/*v*) trichloroacetic acid for 30 min at room temperature. Tissues were washed with phosphate-buffered saline (PBS) and permeabilized by incubation with PBS and 0.2% (w/v) Triton X-100 for 1 h at room temperature. The samples were then blocked with 2% bovine serum albumin in PBS and incubated with rabbit-Vangl1 (Atlas Antibodies #HPA025235) as the primary antibody, followed by labeling with Alexa Fluor 555 donkey anti-rabbit IgG pAb (#A31572) as the secondary antibody. Finally, samples were embedded in Fluoromount-G (SouthernBiotech, Birmingham, AL, USA) and inspected using and an upright microscope (model BX51) equipped with a digital camera. The images were analyzed using Tissue Analyzer software (downloaded from https://grr.gred-clermont.fr). After detecting cells from the staining pattern, the axis of biased staining signals in each cell was evaluated [30]. Rose diagrams were drawn using Octave (https://www.gnu.org/software/octave/).

### Scanning electron microscopy

Samples from rats at 5 days, and at 2, 4, and 6 months after surgery were obtained for scanning electron microscopy (SEM) in the same way as for immunofluorescence microscopy. They were pre-fixed with 2% (*w*/*v*) formaldehyde and 2.5% (w/v) glutaraldehyde in a 100-mM HEPES (4-(2-hydroxyethyl)-1-piperazineethanesulfonic acid) buffer for 2 h at room temperature. After fixation, tissue was dehydrated by ethanol dilution (50%, 60%, 70%, 80%, 90%, 99%, and 100%), immersed in t-butanol, and frozen to − 20 °C before the t-butanol was sublimated off. Samples were then sputter-coated with a platinum-palladium alloy using an ion coater (model IB-3; Eiko, Tokyo, Japan) and inspected using a scanning electron microscope (model S-4700; Hitachi, Tokyo, Japan).

### Transmission electron microscopy

The samples from rats at 2, 4, and 6 months after surgery were obtained and pre-fixed in the same way for transmission electron microscopy (TEM) as they were for SEM. Tissues were dehydrated by ethanol dilution (65%, 75%, 85%, 95%, 99%, and 100%) and propylene oxide, and were embedded in an Epon 812 resin. Ultra-thin sections (70 nm) were cut with a diamond knife and samples were inspected with a transmission electron microscope (model H-7650; Hitachi, Tokyo, Japan). The orientations of the basal feet were analyzed using ImageJ (cells ≥10 and cilia ≥100 per each group). Rose diagrams were drawn using Octave.

## Results

### Epithelial morphology in the inverted tracheal segments

**Fig. 2:**
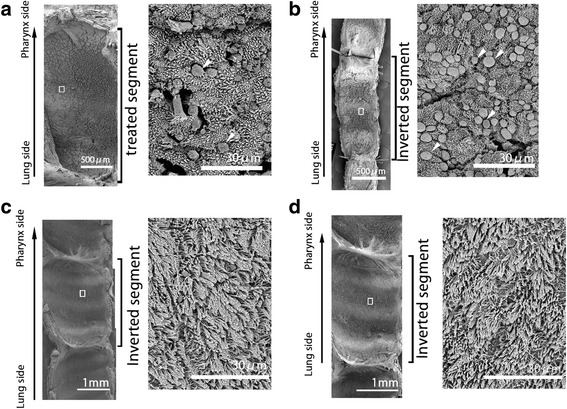
Scanning Electron Microscopy of the Tracheal Epithelia. **a** Five days after surgery in the control group. **b** Five days after surgery in the inverted group. **a**, **b** The epithelium in the treated segment was denatured. Arrowheads indicate disengaging epithelial cells. **c** Two months after surgery in the control group. **d** Two months after surgery in the inverted group. **c**, **d** The ciliated epithelium was well recovered

The morphology of ciliated epithelium was observed by SEM (Fig. 2). Five days after the operation, epithelial cilia were diminished in the inverted trachea and the epithelium had partially degenerated into squamous epithelium in the control and inversion groups. Two months after the operation, however, the epithelium in the inverted segment had recovered its ciliated cells, and was predominantly covered with cilia comparable to that in the normal and treated segments of the control group. The inverted group retained a ciliated morphology at 4 and 6 months after the operation.

### Mucociliary transport direction

To analyze tissue planar polarity in the treated region, mucociliary transport direction was visualized by scattering fluorescent microspheres on the tracheal luminal surfaces. Two months after surgery, the microspheres were transported to the pharyngeal side in the treated region of control rats, similar to that observed in the normal region (Additional file 1: Movie S1); however, the epithelium in the inverted tracheal segments was observed to transport microspheres to the lung side (Additional file 2: Movie S2). Transport to the lung side persisted at 4 and 6 months after inversion (data not shown). For analysis, the pharyngeal and lung directions were defined as 0° and 180°, respectively.**Additional file 1: Movie S1.** Mucociliary Transport in the Control Group. The movement of fluorescent microspheres on the surfaces of normal and treated regions in the control group was captured by high-speed camera connected to a fluorescent microscope at 2 months. Bar = 25 μm. Representative data obtained from three individual experiments are shown. (MP4 5922 kb)**Additional file 2: Movie S2.** Mucociliary Transport in the Inversion Group. The movement of fluorescent microspheres on the surfaces of normal and treated regions in the inversion group was captured by high-speed camera connected to a fluorescent microscope at 2 months. Bar = 25 μm. Representative data obtained from three individual experiments are shown. (MP4 7794 kb)

**Fig. 3:**
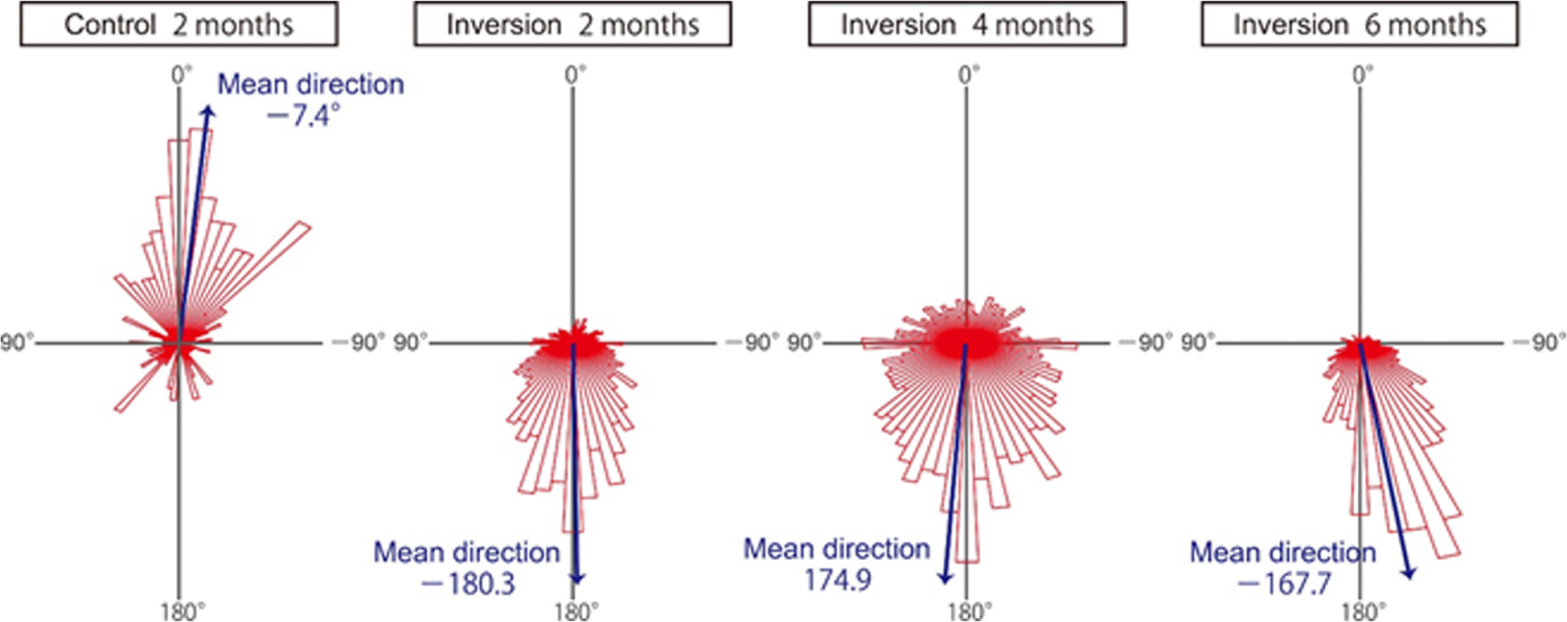
Mucociliary Transport Direction. Pharynx and lung direction were defined as 0° and 180°, respectively. The microsphere movement directions in the auto-transplanted tracheal segments were analyzed. Representative data obtained from individual rats in the control and inversion groups are shown as distribution graphs. Arrows show the mean directions based on the data

Tracking analysis by DIAtrack software showed that transport in the lung direction was marked in the inversion groups at 2, 4, and 6 months postoperatively (Fig. 3). The respective proportions of microspheres moving to directions within 45° of the lung direction (180°) were 65.5% ± 24.6%, 43.4% ± 11.8%, and 53.3% ± 21.5% (Table 1; *n* = 3). The respective mean microsphere transport directions in the inversion group were 177.2°, 175.5°, and 181.5° at 2, 4, and 6 months postoperatively. In contrast to the inversion group, the mean direction in the control group was 3.0° (*n* = 3). There was no significant difference between the percentage of microspheres that moved in the pharyngeal direction in the control group and those that moved in the lung direction in any of the inversion groups (*p* = 0.669). The differences between the percentage of microsphere movements in the lung direction in the control group and those in the pharyngeal direction in any of the inversion groups were also insignificant (*p* = 0.610). The mean direction in the control group had 180° added to and was compared to the mean directions in the inversion groups; however, the values were not statistically different (*p* = 0.876). Therefore, the direction of mucociliary transport was preserved over the long term in inverted tracheae.

**Table 1:** Mucociliary transport direction (*n* = 3)

	Percentage of microspheres		Mean direction
	Within ±45° of pharyngeal direction	Within ±45° of lung direction	
Control, 2 months	63.3% ± 14.9%*	10.5% ± 6.3%^+^	3.0°^#^
Inversion, 2 months	8.6% ± 5.2%^+^	65.5% ± 24.6%*	177.2°^#^
Inversion, 4 months	16.1% ± 7.7%^+^	43.4% ± 11.8%*	175.5°^#^
Inversion, 6 months	14.4% ± 10.7%^+^	55.3% ± 21.5%*	181.5°^#^

*p = 0.669, ^+^*p* = 0.610, ^#^*p* = 0.876

### Ciliary beat direction

**Fig. 4:**
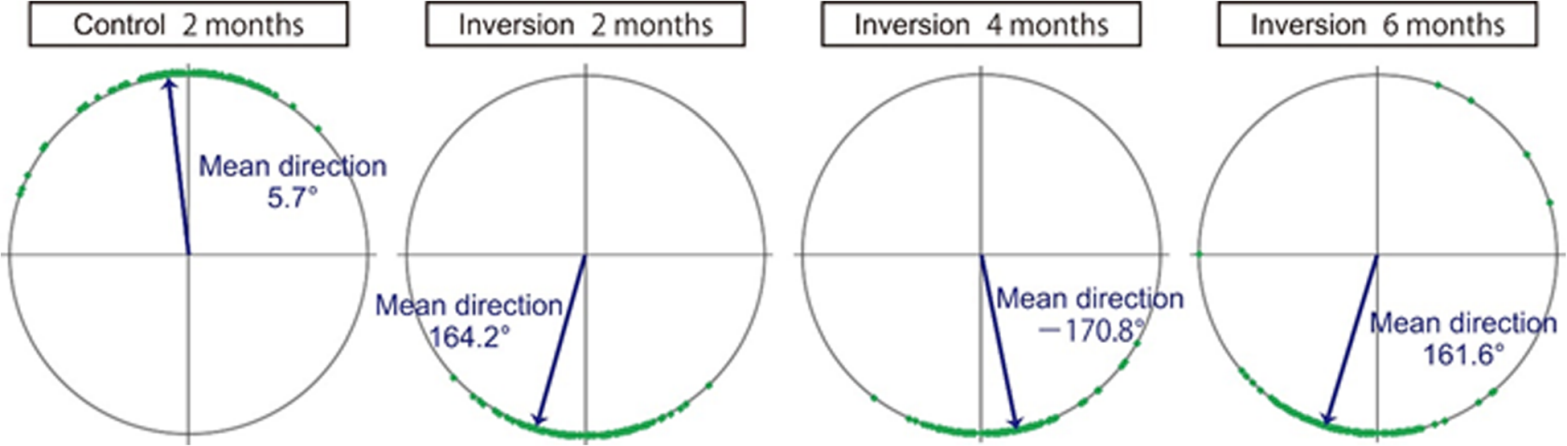
Ciliary Beat Direction. Pharynx and lung direction were defined as 0° and 180°, respectively. The ciliary beat directions in the auto-transplanted tracheal segments were analyzed. Representative data obtained from individual rats in the control and inversion groups are shown. Arrows show the mean directions based on the data

To determine planar polarity at the cellular level, we monitored the direction of ciliary movement in individual cells (more than 100 cells in each rat). Most ciliated cells in the treated and normal regions of the control group showed pharyngeal directed power strokes (Additional file 3: Movie S3). By contrast, the power strokes of ciliated cells in the inversion group were directed toward the lung side at 2 months postoperatively (Additional file 4: Movie S4). The ciliary movement was almost unified within the transplanted segment not only in the central part of the segment but also in parts near the upper and lower anastomosis. Analysis of ciliary beat direction in individual cells revealed that directions were biased toward the lung side at 2, 4, and 6 months after inversion (Fig. 4). The proportions of ciliated cells with ciliary beat directions ranging within 45° of the lung direction (180°) were 97.3% ± 2.1%, 95.3% ± 2.1%, and 95.3% ± 2.5% at 2, 4, and 6 months after inversion, respectively (Table 2; *n* = 3). The mean ciliary beat directions were 177.5°, 178.6°, and 173.6° at 2, 4, and 6 months after inversion, compared with 3.1° in the control group (n = 3). There was no significant difference between the percentage of ciliated cells that beat in the pharyngeal direction in the control group and those that beat in the lung direction in any of the inversion groups (*p* = 0.654). The differences between the percentage of ciliary that beat in the lung direction in the control group and those that beat in the pharyngeal direction in any of the inversion groups were also statistically insignificant (*p* = 0.509). The mean direction in the control group had 180° added to it and was compared to the mean directions in the inversion groups; however, the values were not statistically different (*p* = 0.806). These results show that the direction of ciliary beat was preserved in reversed trachea for long term.**Additional file 3: Movie S3.** Ciliary Beat Direction in the Control Group. The tracheal epithelium in the control group was stained with FITC-WGA at 2 months and the movement of cilia in normal and treated regions was captured by high-speed camera connected to a fluorescent microscope. Bar = 25 μm. Representative data obtained from three individual experiments are shown. (MP4 15123 kb)**Additional file 4: Movie S4.** Ciliary Beat Direction in the Inversion Group. The tracheal epithelium in the inversion group was stained with FITC-WGA at 2 months and the movement of cilia in normal and treated regions was captured by high-speed camera connected to a fluorescent microscope. Bar = 25 μm. Representative data obtained from three individual experiments are shown. (MP4 5922 kb) (MP4 9114 kb)

**Table 2:** Ciliary beat direction (*n* = 3)

	Percentage of ciliated cells		Mean direction
	Within ±45° of pharyngeal direction	Within ±45° of lung direction	
Control, 2 months	95.9% ± 2.9%*	0%^+^	3.1°^#^
Inversion, 2 months	0.7% ± 1.2%^+^	97.3% ± 2.1%*	177.5°^#^
Inversion, 4 months	0%^+^	95.3% ± 2.1%*	178.6°^#^
Inversion, 6 months	0.8% ± 0.8%^+^	95.3% ± 2.5%*	173.6°^#^

*p = 0.654, ^+^*p* = 0.509, ^#^*p* = 0.806

### Positional relationship between the basal body and the basal foot

**Fig. 5:**
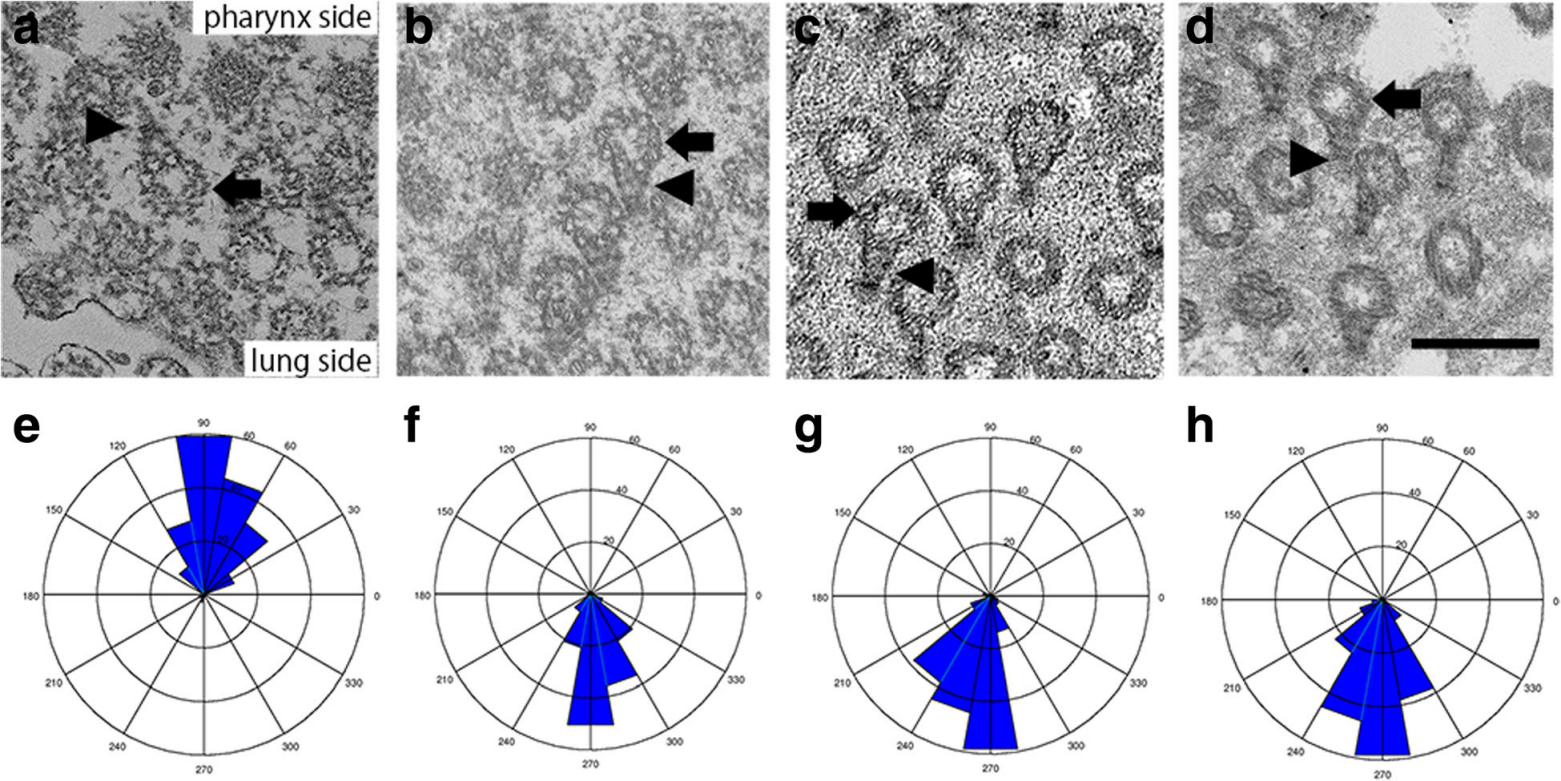
Positional Relationship Between the Basal Body and the Basal Foot. The images in (**a**), (**b**), (**e**) and (**f**) are for the inversion group at 2 months, the images in (**c**) and (**g**) are for the inversion group at 4 months, and the images in (**d**) and (**h**) are for the inversion group at 6 months, and they are orientated so that the upward direction is to the pharynx and the downward direction is to the lung. **a** In the normal segment, the basal foot (arrowhead) of each cilium was on the pharyngeal side of the basal body (arrow). **b**-**d** In the inverted segment, the basal foot (arrowhead) of each cilium was on the lung side of the basal body (arrow). Scale bar = 50 nm. **e**-**h** The distribution graphs of the orientations of the basal feet

The directionality of cilia was further examined by TEM, which showed the positional relationship between the basal body and basal foot (Fig. 5). Although the basal foot was directed toward the pharyngeal side of the basal body in the normal segment, in the inverted segment, it was in the lung side of the basal body 2 months after inversion. The positional relationship between the basal body and basal foot was the same at 4 and 6 months after inversion.

### Evaluation of PCP pathway-related protein localization

**Fig. 6:**
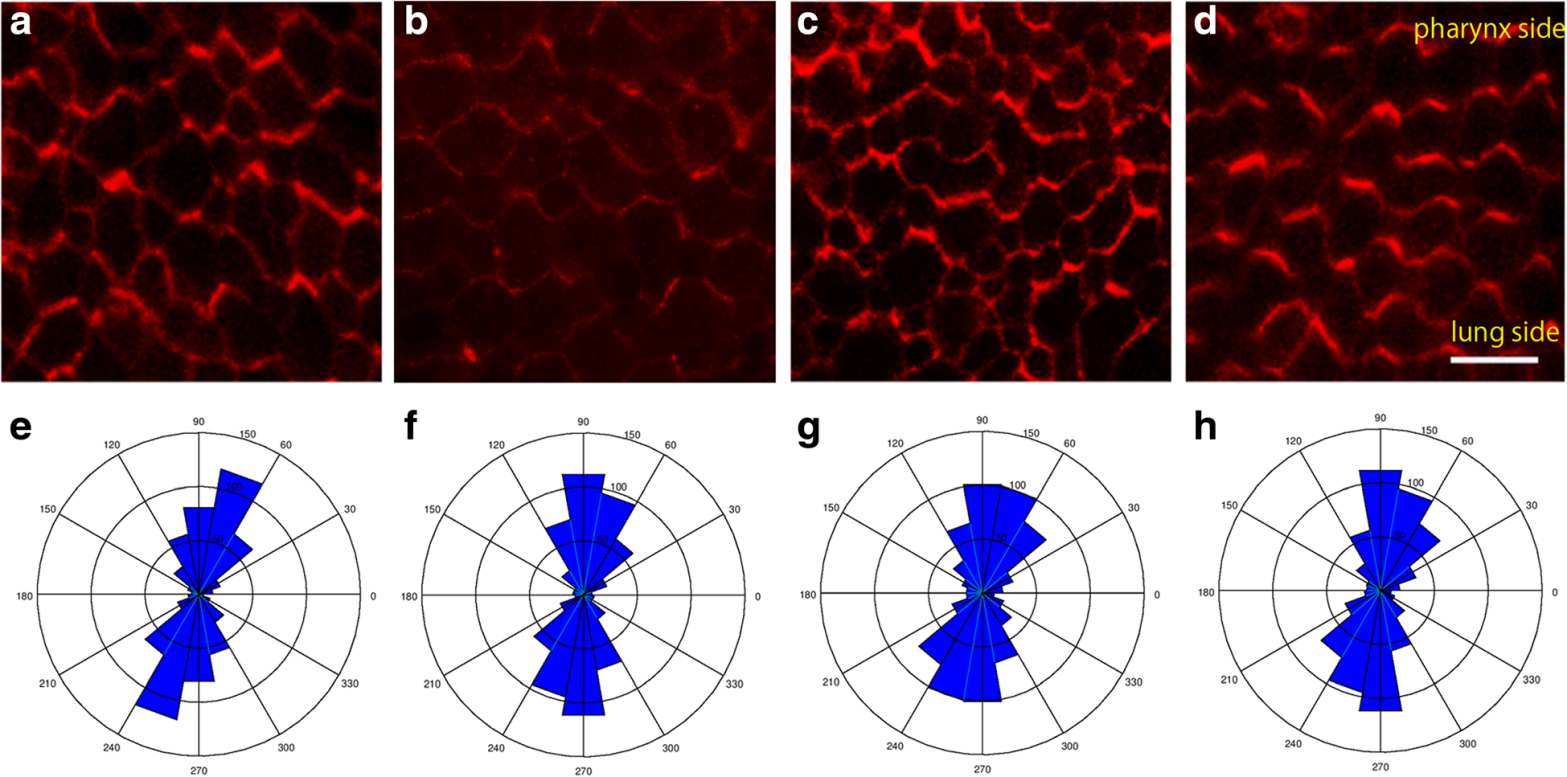
Immunohistochemistry of Vangl1. **a**, **e** Control group at 2 months. **b**, **f** Inversion group at 5 days. **c**, **g** Inversion group at 2 months. **d**, **h** Inversion group at 6 months. **a**–**d** show that staining against Vangl1 was coordinately aligned along the longitudinal axis of the trachea. **e**–**h** show the distribution graphs of axes with no remarkable difference between groups. Scale bar = 10 μm

We also investigated whether the distribution of PCP signaling pathway components, Vangl1, was preserved. Staining against Vangl1 was biased in epithelial cells, and we observed many crescent-shaped structures that coordinately aligned along the longitudinal axis of the trachea (Fig. 6). Moreover, staining against Vangl1 was coordinately aligned along the longitudinal axis of the trachea in all groups. There was no remarkable difference in the distributions of the axis (cells ≥400 per each group) between the groups, suggesting that the PCP along the longitudinal axis was preserved in tracheal epithelial cells 6 months after inversion.

## Discussion

The purpose of this study was to clarify whether orthotropic reversal affected PCP in airway ciliated epithelium. By clarifying this, we have been able to obtain new information on how to achieve the regeneration of ciliated epithelium with normal PCP in reconstructed trachea, based on tissue engineering. This has important implications for whether there is a trend of adjusting the PCP in a desirable direction in an in vivo environment, in other words, whether there is a need to acquire the proper PCP at the time of epithelialization.

Our results showed that, over the long term, the directions of mucociliary transport were maintained in inverted tracheae. The analyses of ciliary beat and basal foot directions also revealed that the long axis was not disturbed, though the local vector was inverted. Moreover, the axis of Vangl1 was not disturbed in the inverted tracheae. Given that the inverted epithelia maintained polarity for the normal turnover time of rat tracheal epithelium, which is reportedly 70–110 days [31], we anticipate that the observed PCP would not be synchronized to the normal region after longer periods than 6 months. Therefore, our data indicate that tracheal tissue holds an in vivo mechanism for reproducing PCP when it has previously been organized, but that this is only in the previously established direction.

External fluid flow has been reported to coordinate the direction of the ciliary movement during the differentiation of stem cells into ciliated cells in *Xenopus* skin and rat ependymal cells [32, 33]. Although there are no reports showing that external fluid flow controls the direction of ciliary movement in airway epithelium, asymmetric Vangl1 crescents are known to disappear at the area without ciliated cells in the sinonasal epithelia of humans with cystic fibrosis or chronic rhinosinusitis. It is speculated that the formation of cilia gives a positive feedback for the establishment of the PCP [16]. In this study, neither obvious respiratory complications nor severe mucus retention were observed after treatment. Therefore, it is considered that the ability to discharge mucus toward the pharynx side is preserved to some extent. Thus, the overall mucus flow, which is induced by respiration or coughing, is assumed not to affect ciliated-cell PCP. PCP maintenance in the tracheal segment was suggested to occur without a polarity cue from the surrounding tissue. This may suggest that the overall flow is not a significant factor in PCP maintenance, and the maintenance depends on the cues encoded in the epithelial and/or subepithelial tissues of the transplanted trachea. On the other hand, at 5 days-post operation in the inverted group, some ciliated cells had survived, and the axis of the epithelial cells in the group was preserved. This suggests that the cilia-generated flow immediately above the epithelial cells in the inverted trachea was maintained to some extent, even after the epithelium was damaged by the operation. There might be a possibility the local liquid flow generated by the remained ciliated cells contributed to maintain airway epithelial PCP. There is a need for further examination to clarify the external factors determining airway epithelial PCP.

As stated, the results of this study show that PCP in the inverted region did not synchronize to that of the normal region. Therefore, to regenerate ciliated epithelium with appropriate planar polarity, the PCP of ciliated epithelia on cellularized scaffold must be properly synchronized in the ciliated cell culture before transplantation. Or, in the case of cell-free scaffolds, the PCP must be adjusted during the epithelial regeneration period perhaps prior to cilia regeneration. It should also be noted that the epithelia grown on artificial scaffolds will lack the underlying tissues. If the cue encoded in subepithelial tissue was important for PCP maintenance, the behavior of the epithelium on artificial scaffolds may be different from the above hypothesis.

Elucidation of the external factors that determine PCP in ciliated cells, and the methods by which this can be controlled, is still needed.

## Conclusion

The PCP of ciliated epithelium was preserved at the tissue and cellular levels in inverted tracheae, even after long-term observation. However, the direction of ciliary movement in inverted tracheae did not synchronize to that of the surrounding normal regions at times exceeding the normal turnover time of rat tracheal epithelium. We conclude that the planar polarity in the inverted segment is not entirely lost in the small segment. PCP maintenance in the tracheal segment was probably done without a polarity cue from the surrounding tissue.

## Abbreviations

FITC-WGA: Fluorescein isothiocyanate-conjugated wheat germ agglutinin
PCP: Planar cell polarity
SEM: Scanning electron microscopy
TEM: Transmission electron microscopy
Vangl: Vang-like
